# Best oral self-care practices for peri-implant conditions and diseases: a systematic review

**DOI:** 10.3389/froh.2025.1657025

**Published:** 2025-12-18

**Authors:** Iwonka T. Eagle, Nicole Theis-Mahon, Michelle C. Arnett

**Affiliations:** 1 Department of Periodontics and Oral Medicine, Division of Dental Hygiene, University of Michigan School of Dentistry, Ann Arbor, MI, United States; 2 Evidence Synthesis Librarian, Health Sciences Collections Coordinator, Health Sciences Library, University of Minnesota, Minneapolis, MN, United States; 3 Department of Primary Dental Care, Division of Dental Hygiene, University of Minnesota School of Dentistry, Minneapolis, MN, United States

**Keywords:** dental implants, peri-implant health, peri-mucositis, peri-implantitis and oral health, prevention and control in oral hygiene

## Abstract

**Introduction:**

The increasing prevalence of dental implants has brought greater attention to the prevention and management of peri-implant diseases, which can compromise long-term implant success. This systematic review evaluated the current evidence on oral self-care practices for maintaining peri-implant health in healthy, non-smoking adults.

**Methods:**

This systematic review was reported in accordance with the PRISMA 2020 statement and registered in the PROSPERO database (CRD420251028140). PICO methods and guidelines for the Center for Evidence-Based Medicine were used to develop the focus question, “*What are the best practices for oral self-care for the prevention and management of peri-implant conditions and diseases?”* Risk of bias was determined by applying the ROBIS Tool to assess risk of bias in systematic reviews and the revised Cochrane risk of bias tool for randomized trials (RoB2).

**Results:**

Across 12 studies, interventions were categorized into five domains: toothbrushes, interdental aids, toothpaste, mouth rinses/topicals, and multi-modal self-care strategies. Powered toothbrushes, particularly oscillating-rotating models, demonstrated superior plaque and inflammation reduction compared to manual options. Triclosan-containing toothpastes consistently outperformed fluoride-only formulations in decreasing plaque, bleeding on probing, and pathogenic bacteria. Interdental aids such as interproximal brushes and oral irrigators were more effective than floss in reducing inflammatory markers. Stannous fluoride-based rinses showed potential anti-inflammatory benefits, while prolonged chlorhexidine use may elevate inflammatory cytokines.

**Discussion:**

A multimodal approach combining mechanical and chemical adjuncts was most effective for peri-implant disease prevention. These findings emphasize the importance of individualized, evidence-based home care protocols in preserving implant longevity and reducing peri-implant disease burden.

**Systematic Review Registration:**

https://www.crd.york.ac.uk/PROSPERO/view/CRD420251028140, PROSPERO CRD420251028140.

## Introduction

Approximately 69% of adults aged 35 or older have experienced the loss of at least one permanent tooth due to periodontal disease, tooth decay, or trauma (1). Implant dentistry, a method for replacing missing teeth, has been utilized for 50 years (2, 3). Over that time, advancements within the field of implant dentistry have led to enhancements in the long-term stability of tooth replacement and fewer complications during and after implant placement (3). Current data suggest that implants have a reliable survival rate of 95% (3, 4). However, the occurrence of peri-implant diseases, such as peri-mucositis (affecting 50% of cases) and peri-implantitis (ranging from 12% to 43% of cases) at the implant site, poses a significant challenge, increasing the risk of implant failure (5, 6). With the growing prevalence of dental implants, the incidence of peri-implant diseases and implant failures has risen accordingly. Peri-implantitis affects approximately 12.5% of implants and 19.5% of patients, while a separate 10-year meta-analysis reported implant survival rates of 93.2%–96.4%, indicating a failure rate of roughly 3.6%–6.8% over that period (7, 8).

Peri-implant health is characterized by the lack of inflammation indicators in the soft tissue around the implant [such as redness, swelling, or excessive bleeding on probing (BoP)], and no further bone loss beyond osseointegration after the implant body was placed (9–12). Healthy peri-implant mucosa typically measures 3–4 mm in height and is either covered by keratinized or non-keratinized tissue (10). The coronal portion of the implant is lined with sulcular epithelium and a thin junctional epithelium (10). Peri-implant mucositis is defined as a reversible condition characterized by BoP, erythema, inflammation, and possible suppuration but no bone loss (9, 10, 13). Peri-implant mucositis occurs when there is an inflammatory lesion lateral to the pocket epithelium that does not go past the pocket epithelium (10). Peri-implantitis presents with the same clinical characteristics as peri-mucositis, but also has progressive crestal bone loss when compared to baseline radiographs (9, 14). In peri-implantitis, the lesions extend apically to the bottom of the pocket epithelium and contain a large amount of plasma cells, macrophages, and neutrophils (10).

Research has pinpointed various risk factors and indicators for peri-implant conditions and diseases. Schwarz et al., after a comprehensive analysis of clinical trials spanning 2003–2017, determined that a history of periodontitis, poor biofilm management, and insufficient implant maintenance are the key contributors to the onset and progression of peri-mucositis and peri-implantitis (15). This evidence warrants an investigation into current studies on oral self-care for biofilm removal for peri-implant conditions and diseases. The research question for this systematic review (SR) is, “What are the best practices for oral self-care for the prevention and management of peri-implant conditions and diseases?”

## Methods

This systematic review was reported in accordance with the Preferred Reporting Items for Systematic reviews and Meta-Analyses (PRISMA) 2020 statement (16). The SR protocol was registered in the PROSPERO database (CRD420251028140) (17). The PICO method and the Center for Evidence-Based Medicine guidelines (18) were used to develop the focus question, “What are the best practices for oral self-care for the prevention and management of peri-implant conditions and diseases?”

### PICO

The population (P) of adult patients in this SR was those 18 years or older with a dental implant, not affected by systemic conditions (i.e., diabetes, autoimmune diseases), cancers, HIV/AIDs, or any form of tobacco use. The intervention (I) was oral self-care practices with no comparison (C). The outcome (O) was the prevention and management of peri-implant conditions and diseases.

### Eligibility criteria

The included studies were United States (US) and international peer-reviewed articles, studies with randomized research designs, clinical trials, observational studies, meta-analysis, systematic reviews, and reviews that included at least one keyword from the “oral self-care practices” or “outcomes” category. Letters, editorials, or comments, cohort studies, and case studies were excluded. Moreover, studies with unhealthy patients/populations with an implant [systemic conditions (i.e., diabetes, autoimmune diseases), smokers, cancers, HIV/AIDs] and those that included professional prevention or management of a peri-implant disease or condition from a licensed oral health profession [i.e., non-surgical periodontal therapy (NSPT) (i.e., hand instrumentation, ultrasonic scaling, or piezo scaling), air-polishing, locally delivered antibiotics, irrigation (chairside irrigation), or surgical periodontal therapy (i.e., implantoplasty/gingival or bone graft) were also excluded.

### Search and screening

A search was conducted using a combination of controlled vocabulary and natural language keywords. The search was designed by a health science librarian (NT-M) and included the concepts of peri-implants and oral self-care practices (i.e., interdental aids, dental floss, manual and electronic toothbrushes, therapeutic irrigation, and toothpaste), and periodontal indices {[i.e., Silness and Löe indices [gingival (GI) and plaque indices (PI)]} and plaque scores [O’Leary plaque score and Quigley and Hein index (later modified by Turesky et al.)].

A search was developed for Medline(R)ALL and then translated and executed across five additional databases: Embase + Embase Classic (via Ovid), Cinahl, Dentistry and Oral Sciences Source (EBSCO), Scopus, and Web of Science Core Collection (SCI-EXPANDED, SSCI, AHCI, CPCI-S, CPCI-SSH, BKCI-SSH, ESCI, CCR-EXPANDED, and IC) from their inception to 16 April 2025 (Appendix with Search Strategies). The results were limited to studies on humans and in English, and excluded letters, commentary, and editorials.

Results were then exported to Covidence for deduplication and screening against the inclusion and exclusion criteria (19). The two co-primary investigators (PIs) independently and blindly screened titles and abstracts against the inclusion criteria. A virtual Zoom (Zoom; San Jose, CA, USA) meeting occurred on 14 May 2025 for the two co-PIs to discuss conflicts and undetermined articles until an agreed consensus was achieved. The full-text articles were then reviewed and 12 met the exclusion criteria.

### Risk of bias

The risk of bias in systematic reviews (ROBIS) tool (20) was used to assess the risk of bias in four systematic reviews (*n* = 4) (21–24). The tool is implemented in the following three phases: (1) assess the review's relevance, (2) identify potential issues in the review process, and (3) evaluate the risk of bias (20). In phase 2, the following four key areas where bias may occur in a systematic review are examined: study eligibility criteria, how studies were identified and selected, data collection and appraisal methods, and the synthesis and interpretation of findings. Phase 3 involves judging the overall risk of bias in how the review findings are interpreted, considering any limitations flagged in phase 2 (20).

The revised Cochrane risk of bias tool for randomized trials (RoB2) (25) was used for nine studies (*n* = 8) (26–33). The five domains, namely, (1) bias arising from the randomization process, (2) bias due to deviations from intended interventions, (3) bias due to missing outcome data, (4) bias in measurement of the outcome, and (5) bias in selection of the reported result, were rated either (a) low risk of bias, (b) some concerns, or (c) high risk of bias (25).

## Results

### Studies included

**Figure 1:**
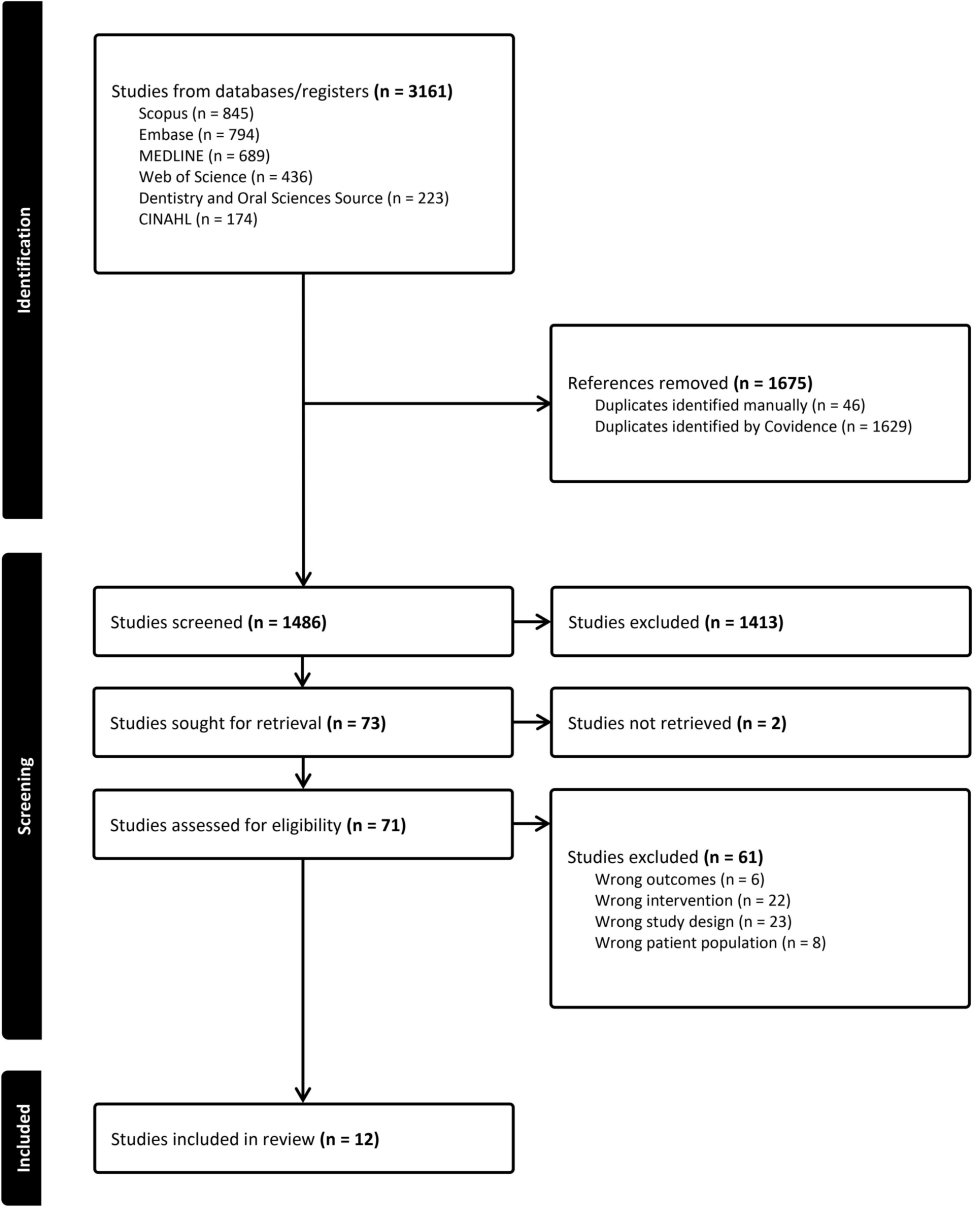
PRISMA flowchart.

The search yielded a total of 3,161 articles with 1,486 remaining after the duplicates were removed (Figure 1). The search and selection process is outlined in Figure 1. The articles in this systematic review were categorized into the following five domains: (1) toothbrushes, *n* = 3; (2) interdental aids, *n* = 4; (3) toothpaste, *n* = 3; (4) mouth rinses and topicals, *n* = 1; and (5) multiple oral self-care interventions, *n* = 1. The domains were further separated into the following categories: toothbrushes: (a) manual and (b) electric; interdental aids: (a) floss, (b) interdental brushes, and (c) oral irrigation; toothpaste: (a) fluoride toothpaste and (b) triclosan toothpaste; mouth rinses and topicals: (a) chlorhexidine (CHX) and (b) essential oils.

### Study characteristics

Table 1 presents the study characteristics. In the 12 studies in this systematic review, adult patients at least 18 years of age or older were included (21–24, 26–33). The majority of the studies included only healthy, non-smokers, with at least one dental implant (26–28, 31–33). One study did not disclose the health or smoking status of the participants (29). The SRs and narrative reviews also only included adults who were in good general health and non-smokers (23). Two SRs (21, 24) and one review of the literature (22) did not mention the inclusion criteria for the participants. Two double-blind randomized controlled trials (RCTs) with parallel groups (26, 27), one single-blind three-group parallel RCT (33), two single-examiner-masked RCTs (31, 32), two RCTs (28, 30), one longitudinal multicenter RCT (29), two SRs (21, 24), one literature review (22), and one narrative review (23) were included.

**Table 1:** Study characteristics.

Domain and categories	NLM citation	Research design	Purpose/aim(s), sample size/population	Outcome measures	Results	Strengths and limitations
**Domain**: toothpaste **Categories**: triclosan and fluoride dentifrices	Ramberg P, Lindhe J, Botticelli D, Botticelli A. The effect of a triclosan dentifrice on mucositis in subjects with dental implants: a six-month clinical study. *J Clin Dent*. 2009;20 (3):103–7.	Double-blind, randomized, two-treatment, parallel-group clinical study	This 6-month RCT assessed the efficacy of dentifrice containing triclosan on peri-implant mucositis Intervention group: 0.3% triclosan and 2.0% polyvinylmethyl ether/maleic acid copolymer in a sodium fluoride silicon-based dentifrice Control group: 0.243% sodium fluoride dentifrice Sample: *n* = 59 adult patients in good general health, with documented tooth loss to periodontitis and at least two dental implants; one dental implant with peri-implant mucositis	Outcome measures collected at BL, 3 months, and 6 months Outcomes measures were PD, BoP, and plaque	Probing depth: The test group had a statistically significant reduction at 3 and 6 months (*P* < 0.001) Bleeding on probing was statistically lower in the test group at 3 and 6 months (*P* < 0.001) Plaque: The mean reduction between the BL and 3 months was statistically significant in both groups (*p* < 0.001). There was no difference between the groups at 6 months	Strengths: double-blind and parallel-group research design Limitations: No reporting guidelines or registries noted
**Domain**: toothpaste **Categories**: triclosan and fluoride-containing dentifrices	Sreenivasan PK, Vered Y, Zini A Mann J, et al. A 6-month study of the effects of 0.3% triclosan/copolymer dentifrice on dental implants. *J Clin Periodontol*. 2011 Jan;38 (1):33–42.	Double-blind, randomized, two-treatment, parallel-group clinical study	This 6-month RCT examined the effect of a dentifrice containing triclosan on oral biofilms and gingival inflammation in dental implant and peri-implant tissues Sample: *n* = 120 adult patients in good general health, with at least 20 natural teeth and one dental implant without systemic disease or smoking. *n* = 60 assigned to the 0.3% triclosan and 2.0% PVM/MA copolymer in a sodium fluoride silicon base dentifrice *n* = 60 assigned to the 0.243 sodium fluoride dentifrice	The outcome measures, i.e., plaque indices and BoP, were collected at BL, 3 months, and 6 months The outcome measures were modified dental PI for dental implants, Löe and Silness GI on natural teeth, modified SBI, PD, and gram-negative anaerobes assay	Plaque: Both the modified dental PI and Löe and Silness PI were statistically significant (*P* < 0.05) in the 0.3% triclosan group BoP: Statistically significant reductions at 3 and 6 months (*P* < 0.05) Gram-negative anaerobes assay: There were significantly fewer Gram-negative anaerobes in the triclosan group (*P* < 0.05), including 490% reductions in *Aggregatibacter actinomycetemcomitans*, *Campylobacter rectus*, *Eubacterium saburreum*, *Fusobacterium nucleatum*, *Porphyromonas gingivalis*, *Prevotella melaninogenica*, *Solobacterium moorei*, and *Tannerella forsythia*	Strengths: double-blind and parallel-group research design and ethics committee review. The assay supports the inclusion of microbial data Limitations: Conflict of interest as the authors were employees of the sponsor of the study
**Domain**: toothpaste **Categories**: triclosan dentifrice and sodium fluoride and monofluorophosphate dentifrices	Trombelli L, Farina R. Efficacy of triclosan-based toothpastes in the prevention and treatment of plaque-induced periodontal and peri-implant diseases. *Minerva Stomatol*. 2013 Mar;62 (3):71–88.	Systematic review with five focused questions Focus question 5 addressed the impact of triclosan toothpaste on peri-implant disease	Determine the efficacy of a dentifrice containing triclosan on periodontal and peri-implant diseases compared to sodium fluoride and monofluoride phosphate dentifrices	Study characteristics, main results, clinical results, and microbial results	Focus question 5: (*n* = 2) two double-blind parallel-arm RCTs reported greater reductions in plaque and gingival inflammation (BoP and PD) in the triclosan groups	Strengths: None reported Limitations: No registry of systematic review protocol reported. One screener
**Domain**: mouth rinse **Categories**: stannous fluoride CHX	Di Carlo F, Quaranta A, Di Alberti L, et al. Influence of amine fluoride/stannous fluoride mouthwashes with and without chlorhexidine on secretion of proinflammatory molecules by peri-implant crevicular fluid cells. *Minerva Stomatol*. 2008 May;57 (5):215–21; 221–5.	Randomized controlled trial	To evaluate the influence of amine fluoride/stannous fluoride (AmF-SnF_2_) vs chlorhexidine 0.12% (CHX) combined with Am-SnF_2_ on IL-1β, PGE_2_, and EGF secretion by cells in crevicular peri-implant fluid Included a total of *n* = 30 generally healthy adults Control: CHX rinsing during the first 7 days and AmF-SnF_2_ during the following 7 days. Test Group: AmF-SnF(2) rinsing for 14 days	GCF samples: Levels of IL-1β, PGE_2_, and VEGF in the samples were determined using an enzyme-linked immunosorbent assays (ELISA) kit	The AmF-SnF_2_ group had very low levels of IL-1β and VEGF after a 2-week period. PGE_2_ slowly decreased after 2 weeks, possibly due to the short duration of the study. The CHX group showed a higher result for all cytokines The use of Am-SnF_2_ mouthwashes could lead to decreased levels of IL-1β, PGE_2_ and VEGF that are normally stimulated and increased during inflammation events	Strengths: Short duration. Patient demographics not listed Limitations: No reporting guidelines or registries noted
**Domain**: interdental aids **Category:** interproximal brushes, Oral irrigation, and Dental floss	AlMoharib HS, Al Askar MH, Abuthera EA, Alshalhoub KA, et al. Efficacy of three interdental cleaning methods for peri-implant health maintenance of single implant supported crowns: A randomised clinical trial. *Oral Health Prev Dent*. 2024 Jan 15:22:51–56.	A single-blinded, three-group, parallel randomized clinical trial	The purpose was to investigate the effectiveness of interproximal brushes, water flossers, and dental floss to reduce plaque and inflammation A sample of *n* = 45 healthy adult, non-smoking patients with at least one implant in the mandibular posterior region over a 2-week period	Silness and Löe PI Interleukin (IL)-6 using ELISA for PICF	All three interdental aids reduced PI score at the conclusion of the 2 weeks Interproximal brushes yielded statistical improvements in IL-6 levels (*P* = 0.008) compared to dental floss and water flossers	Strengths: None reported Limitations: Sample size may have limited the detection of smaller differences in IL-6 levels and the short study duration (2 weeks). Participants self-reported their adherence to their allocated interdental aid. No reporting guidelines or registries were noted
**Domain**: interdental aids **Category:** dental floss and interdental brushes	Gandhi G, Masanam BSL, Nair AS, Semani N, et al. Efficacy of oral irrigators compared to other interdental aids for managing peri-implant diseases: a systematic review. *BDJ Open*. 2025 Jan 29;11 (1):7.	Systematic review	The purpose was to compare the efficacy of home use of oral irrigators compared to other mechanical plaque control methods (floss/interdental brushes) for clinical indicators of peri-implant diseases. A total of 7 studies met the inclusion criteria	RoB2 ROBINS-I BoP GI, PD, and CAL	ROB2: Four studies were found to have a low risk of bias, one study had some concerns, and two studies had a high risk of bias Oral irrigators, when paired with a manual toothbrush, were found to be 2.45-fold (145%) more effective than flossing in reducing bleeding around implants and PI score and BoP were significantly lower (*P* < 0.05) No significant difference in BoP at the end of the 12 weeks Implant sites where an oral irrigator was used showed a greater reduction in BoP (81.8% vs 33.35%) compared to sites where the floss was used (*P* = 0.0018). The levels of red and orange complex bacteria in the peri-implant biofilm were lower with the use of an oral irrigator than with toothbrushing alone	Strengths: PRISMA checklist, protocol registered in the International Prospective Register of Systematic Review PROSPERO (CRD42023469319) Limitations: Limited number of studies and only a maximum of 6 months of follow-up. No studies on microbial profile, marginal bone levels, or osseointegration
**Domain**: interdental aids **Categories**: oral irrigation and dental floss	Magnuson B, Harsono M, Stark PC, Lyle D, et al. Comparison of the effect of two interdental cleaning devices around implants on the reduction of bleeding: a 30-day randomized clinical trial. *Compend Contin Educ Dent*. 2013 Nov-Dec:34 Spec No 8:2–7.	Randomized single-examiner-masked, single-center study	The purpose of this 30-day study was to determine the effectiveness of water flossers in reducing BoP compared to flossing Adult healthy, non-smoking patients (*n* = 30) with at least two of six sites around an implant with BoP were included	BoP	No significance in BoP between the groups (*P* = 0.2655)	Strengths: None reported Limitations: No reporting guidelines or registries noted
**Domain**: interdental aids **Categories**: oral irrigation and dental floss	Mahajani MJ, Kalla M, Sonkesriya S, Mehra P, et al. Comparison of the impact of two interdental cleaning devices on the reduction of bleeding around implants. *J Pharm Bioall Sci*. 2024;16:S192–5.	Randomized single-examiner-masked, single-center study	The purpose of this 30-day study was to determine the effectiveness of water flossers in reducing BoP compared to flossing Adult healthy, non-smoking patients (*n* = 40) with at least two of six sites around an implant with BoP were included	BoP	The water flosser group had a threefold difference in BoP (*P* < 0.001)	Strengths: No reported Limitations: No reporting guidelines or registries noted
**Domain**: multiple oral self-care interventions **Categories**: manual vs powered toothbrush Powered toothbrush Homecare maintenance Interproximal brushes Superfloss Mouth rinse CHX	Checchi V, Racca F, Bencivenni D, et al. Role of dental implant homecare in mucositis and peri-implantitis prevention: a literature overview. *Open Dent J*, 2019;13	Literature review Question: “Is there scientific evidence reported in the literature that we are using the proper hygiene tools or antimicrobials for dental implant rehabilitation homecare to prevent mucositis and peri-implantitis?”	To identify the presence of scientific evidence that peri-implant home care plays a role in mucositis and peri-implantitis prevention A total of 7 studies were included (three RCTs, one consensus report, one cohort study, one systematic review, and one review) Fourteen other studies that partially met the inclusion criteria were analyzed and classified into the following three levels of evidence: good evidence for RCTs, fair evidence for case control and cohort studies, and poor evidence for expert opinion and case reports	RCT and COHORT studies with 6 months follow-up (*n* = 20) Outcomes: PD, PI, BI, and BoP Consensus paper, systematic review/review, and case control study outcomes: PI and PD	CHX gel (*n* = 6) decreased BI, BoP and PD and reduced edema in antiphlogistic activity Other mouth rinse agents seemed to be effective in reducing BI, GI and inhibiting plaque formation One 12-month cohort study (*n* = 100) found that powered toothbrushes significantly reduced PD, REC, and bleeding score compared to manual toothbrushes A single-blind RCT (*n* = 40) reported no statistically significant difference in PI or BI between brushing methods Interdental devices were reported to reduce PI and BoP Superfloss could be a risk factor for peri-implantitis One systematic review of nine studies concluded that there was little evidence of what the best practices were	Strengths: None reported Limitations: No reporting guidelines or registries noted
**Domain**: toothbrush **Categories**: Counter-rotational powered brush (CRPB) Interplak power toothbrush vs. manual toothbrush	Truhlar RS, Morris HF, Ochi S. The efficacy of a counter-rotational powered toothbrush in the maintenance of endosseous dental implants. *J Am Dent Assoc*. 2000 Jan;131 (1):101–7.	Longitudinal, multicenter, randomized, clinical study	The purpose was to compare the effectiveness of a counter-rotational powered toothbrush compared to a manual toothbrush on indices of periodontal health and implant survival at 24 months after uncovering the implants Group 1: Conventional manual toothbrush Group 2: manual methods plus twice-daily 0.12 percent chlorhexidine rinses (Peridex, Zila Pharmaceuticals Inc., Phoenix, Arizona) for the duration of the study Group 3: CRPB (Interplak Power Toothbrush, Conair Corp., Phoenix, Arizona) Group 4: CRPB plus the same twice-daily chlorhexidine rinses as used by Group 2 The use of a chlorhexidine rinse was assigned randomly to half of the hospitals in each of the CRPB and manual toothbrush groups	Silness and Löe PI Löe and Silness GI CI CAL, PD, and REC Implant survival at the 24-month follow-up visit	The use of a counter-rotational powered toothbrush was significantly different (*P* < 0.001) in plaque removal and improved GI measures	Strengths: Industry sponsorship, investigator training and consistency, and patient reporting and compliance Limitations: None reported. No reporting guidelines or registries noted
**Domain**: toothbrush **Categories**: rotating-oscillating head vs. sonic action head on powered toothbrush.	Preda C, Butera A, Pelle S, Pautasso E, et al. The efficacy of powered oscillating heads vs. powered sonic action head toothbrushes to maintain periodontal and peri-implant health: a narrative review. *Int J Environ Res Public Health*. 2021 Feb 4;18 (4):1468.	Narrative review	To compare the efficacy of rotating-oscillating head (ORH) vs. sonic action head (SAH) powered toothbrushes for plaque accumulation and gingival inflammation In total, 12 trials (*n* = 1,433 participants) were included. The differences between ORH and SAH toothbrushes were expressed as weighted mean differences (WMDs) and 95% confidence intervals	PI GI RoB	Plaque removal: Both ORH and SAH toothbrushes demonstrated comparable efficacy in reducing plaque levels Gingival inflammation: short-term studies (up to 3 months) with unclear risk of bias indicated a significant reduction in gingival inflammation when using ORH toothbrushes Longer-term studies (up to 6 months) with varying risk of bias suggested that SAH toothbrushes were more effective in reducing gingival inflammation over time	Strengths: Heterogeneity in study design Limitations: short follow-up period, risk of bias, different indices, lack of standardization, and underrepresentation of peri-implant outcomes. No reporting guidelines or registries noted
**Domain**: toothbrushes **Categories**: rotating-oscillating head Manual toothbrush	Allocca G, Pudylyk D, Signorino F, Grossi GB, Maiorana C. Effectiveness and compliance of an oscillating-rotating toothbrush in patients with dental implants: a randomized clinical trial. *Int J Implant Dent*. 2018 Dec 10;4 (1):38.	Randomized clinical trial	To assess the efficacy of an oscillating-rotating toothbrush in reducing plaque and inflammation around dental implants Healthy non-smoking patients (*n* = 80) with dental implants were enrolled in this study Test group: *n* = 40, oscillating-rotating toothbrush (one toothbrush head for implants and one for natural teeth) Control: *n* = 40, manual toothbrush	PI BoP PPD Baseline and 1-month and 3-month follow-ups	PI and BoP were statistically significantly different for both the test and control groups (*P* < 0.0001) Implant sites showed higher values for both BoP and PI An oscillating-rotating toothbrush was effective in reducing new plaque formation (*P* < 0.0001) and bleeding (*P* < 0.0001) at the implant site and dental sites compared to manual ones (*P* > 0.05) at 1 and 3 months No significant differences in PPD	Strengths: None reported Limitations: Short follow-up duration, monocentric design, Hawthorne effect (potential), and specific toothbrush models used No reporting guidelines or registries noted

RCT, Randomized control trial; BL, baseline; PD, probing depth; CAL, clinical attachment loss/level; REC, recession; BoP, bleeding on probing; PI, plaque index; GI, gingival index; B, bleeding index; SBI, sulcus bleeding index; CI, calculus index; CHX, chlorhexidine; ROB2, Cochrane risk of bias assessment tool; ROBINS-I, risk of bias in non-randomized studies of interventions.

The intervention durations were 6 months for two studies (26, 27), 3 months for one study (28), 1 month for three studies (31, 32), and 2weeks for two studies (30, 33). One study was longitudinal, with a duration of 24 months (29). The interventions were triclosan- and fluoride-containing dentifrices in two studies (26, 27) and one SR focused on the effectiveness of triclosan-containing dentifrices in peri-implant diseases (21). Mouthwashes or gels containing CHX or stannous fluoride were the interventions in one study (30). Interdental aids, namely, interproximal brushes, oral irrigation, or dental floss, were the interventions in three (31–33) and there was one SR focused on these interventions (24). Moreover, toothbrushes, including oscillating, sonic, or manual toothbrushes, were the interventions in two studies (28, 29). One literature review included three domains from this SR (toothbrushes, interdental aids, and mouthwash) (22).

The clinical measures reported were probing depth (PD) (22, 24, 27–29, 31, 32), recession (REC) (29), clinical attachment level (CAL) (24, 29), BoP or sulcular bleeding index (SBI) (22, 24, 26–28), Löe and Silness PI (22, 23, 26–29, 33), or GI (23, 24, 26). The calculus index (CI) was reported in one study (29) and microbial assay or peri-implant crevicular fluid (PICF) was reported in four studies (21, 26, 30, 33). A risk of bias in reporting was noted in two studies (23, 24). The strengths of these studies are presented in Table 1. The observed limitation in a number of the studies was the lack of reporting guidelines or registries (21–23, 27–33).

### Quality (risk of bias) assessment

Of the 12 articles in this systematic review, four were evaluated using the ROBIS tool (Table 2). In phase 1, we assessed the relevance of the studies, including the patients/population, interventions, comparators, and outcomes (Table 1) (21–24). One SR had a high risk of bias due to the total number of focused research questions (five), having limited key words (two total), and having only one screener for the inclusion and exclusion criteria (21). The SR by Gandhi et al. was deemed to have a low risk of bias for all domains in the ROBIS tool (24). The SR by Preda et al. generally had a low risk of bias, with the exception in phase 3 due to only having one reviewer (23). The SR by Checchi et al. covered a wide range of oral hygiene practices and was deemed low risk of bias for domains 1 and 2 in phase 2, but was deemed to have a high risk of bias for domain 3 in phase 2 (22). This high risk of bias was determined because no risk of bias validation tool was used and the authors categorized evidence against the inclusion criteria, which may have bias (22).

**Table 2:** Results from the ROBIS tool: Low = 1, high = 2, unclear = ?.

Author	Phase 2				Phase 3
	1: Study eligibility criteria	2: Identification and selection of study	3: Data collection and study appraisal	4: Synthesis and findings	Judging risk of bias
Trombelli, 2013	?	2	2	?	2
Checchi, 2019	1	1	2	?	?
Preda, 2021	1	1	2	1	?
Gandhi, 2025	1	1	1	1	1

A total of eight studies were evaluated using RoB2 (Table 3). For domain 1, i.e., the risk of bias arising from the randomization process, five studies had a low risk (26, 28, 30–32) and two had some concerns (27, 28). For domain 2, i.e., the risk of bias due to deviations from the intended interventions, two studies had a low risk (26, 27) and five studies had some concerns (28–32). For domain 3, i.e., missing outcome data, five studies had a low risk (26–28, 31, 32) and two studies had some concerns (29, 30). For domain 4, i.e., risk of bias in measurement of the outcome, five studies had a low risk (26, 27, 29, 31, 32) and two studies had some concerns (28, 30). For domain 5, i.e., risk of bias in the selection of the reported results, four studies had low risk (26, 28, 31, 32) and three studies had some concerns (27, 29, 30). All the studies assessed using RoB2 had some concerns overall (27–32), except one, which had a low risk of bias overall. A quantitative synthesis was not performed due to wide heterogeneity among studies in terms of study design, intervention and comparator groups, and study duration.

**Table 3:** Results from RoB2.

Author	D1	D2	D3	D4	D5	Overall
Sreenivasan, 2011	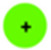	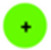	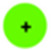	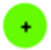	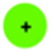	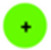
Ramberg, 2009	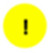	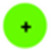	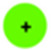	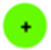	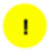	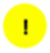
Allocca, 2018	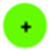	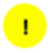	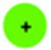	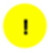	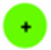	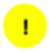
Truhlar, 2000	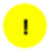	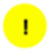	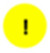	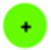	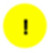	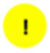
Di Carlo, 2008	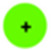	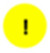	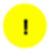	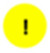	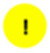	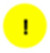
Mahajani, 2024	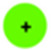	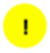	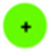	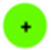	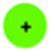	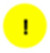
Magnuson, 2013	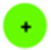	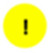	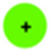	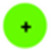	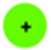	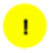
AlMoharib, 2024	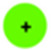	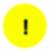	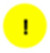	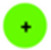	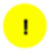	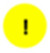

Domains: D1 = randomization process, D2 = deviations from the intended interventions, D3 = missing outcome date, D4 = measurement of the outcome, D5 = selection of the reported results. High risk, 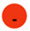 some concern, 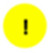 low risk, 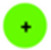.

## Discussion

Poor biofilm management through oral self-care remains a key factor in the onset and progression of peri-mucositis and peri-implantitis (15). The goal of this systematic review was to identify the optimal home care devices, aids, or adjuncts to reduce plaque and inflammation, with no professional intervention. The overarching findings were that triclosan-containing toothpaste consistently reduced plaque, BoP, and pathogenic bacteria, with superior performance over fluoride-only toothpaste (21, 26, 27). Interdental aids, particularly interproximal brushes and oral irrigators, were more effective than floss in reducing BoP and inflammatory markers (22, 24, 31–33). Oscillating power toothbrushes were more effective than manual toothbrushes in reducing PI score and BoP, and limited clinical differences were reported for PD reduction (22, 23, 28, 29). Mouth rinses with stannous fluoride had anti-inflammatory effects; however, it was reported that those with CHX had mixed results and the potential to increase cytokine levels with prolonged use (30).

### Toothpaste

Clinical trials consistently support the use of triclosan-containing dentifrices (0.3% triclosan with 2.0% polyvinylmethyl ether (PVM)/maleic acid (MA) copolymer and fluoride) in reducing plaque, BoP, and PD around dental implants (21, 26, 27). Compared to standard sodium fluoride toothpaste, triclosan formulations show statistically significant reductions in gingival inflammation and microbial load, including periodontal pathogens such as *Porphyromonas gingivalis* and *Tannerella forsythia* (26). These findings support Ribeiro et al.’s investigation of triclosan-containing toothpaste as a protective approach to prevent peri-implant tissue inflammation (34). Interestedly, Ribeiro et al. identified reduced inflammation measures and BoP, even in the presence of biofilm (34). A triclosan-containing toothpaste may help reduce inflammation and support the host response around peri-implant tissue, even without standard oral hygiene practices for biofilm management (21, 26, 27, 34). This evidence suggests triclosan-containing toothpaste is a valuable adjunct to consider as a best practice for preventing peri-implant diseases due to its anti-inflammatory properties, which may potentially decrease the need for more invasive interventions. Based on the evidence, dental professionals should consider recommending triclosan-containing toothpaste as part of a comprehensive peri-implant maintenance program while also emphasizing proper brushing techniques to maximize its benefits. Regular clinical assessments should also be performed to evaluate the impact of triclosan toothpaste on peri-implant soft tissue health to further help guide personalized oral hygiene strategies.

### Mouth rinses

The use of mouth rinses has long been indicated as an adjunct to traditional tooth brushing and flossing for control of peri-implant biofilm. Traditionally, 0.12% chlorhexidine digluconate and essential oils have both been shown to be clinically effective in the prevention of peri-implant disease by inhibiting and reducing the formation of biofilm (35). Though historically used, a randomized clinical study now indicates that chlorhexidine may elevate inflammatory markers; therefore, long-term use should be approached cautiously due to its potential side effects (30). Interestingly, stannous fluoride/amine fluoride rinses, particularly when used without adjunctive chlorhexidine, specifically demonstrated potential in reducing inflammatory cytokines such as interleukin 1β (IL-1β) and vascular endothelial growth factor (VEGF) in peri-implant crevicular fluid. While only one mouth rinse study was included in this systematic review, this evidence strongly indicates that stannous fluoride/amine fluoride rinses should be considered as an alternative for peri-implant health. The potential anti-inflammatory benefits of stannous fluoride/amine fluoride rinses warrant consideration in patients, especially in those with early signs of peri-implant inflammation or those at risk for biofilm accumulation. Future recommendations should prioritize these formulations over chlorhexidine for routine use, reserving chlorhexidine for short-term, targeted interventions. It is vital that dental professionals provide clear patient education on proper adjunctive rinse use to sustain peri-implant health.

### Interdental aids

Biofilm removal between implants is vital and interproximal brushes showed superior outcomes in reducing the clinical indicators of peri-implant diseases and inflammatory markers (e.g., IL-6) compared to dental floss and water flossers (22, 24, 31–33). Nevertheless, oral irrigators, especially when combined with toothbrushing, significantly reduce BoP and the presence of pathogenic bacteria compared to flossing alone (33) and were reported as an appropriate adjunct to toothbrushing for the management of peri-implant tissues (24). It is important to note that the findings from this systematic review support the inclusion of interdental aids as a best practice for the prevention and management of peri-implant diseases; however, patient preferences, dexterity, and implant configuration should be considered when making professional recommendations for oral self-care strategies.

### Toothbrushes

Patients with dental implants often have unique needs based on their implant location and prosthetic design. Choosing the right toothbrush tailored to those needs ensures more effective maintenance and better outcomes. Powered toothbrushes, including oscillating-rotating and counter-rotating models, are more effective than manual toothbrushes at reducing plaque and gingival inflammation (29). Studies show that oscillating-rotating heads may offer better outcomes at implant sites than sonic brushes in the short term, although long-term benefits are comparable (23, 28). These devices also enhance patient compliance and ease of use, which are critical in peri-implant health. Clinically, these findings highlight the importance of individualized oral hygiene instruction and toothbrush device selection for implant patients. The superior short-term plaque control observed with oscillating-rotating powered brushes supports their recommendation as a home care tool, particularly for individuals with limited dexterity or complex prosthetic designs. Toothbrush recommendations should be tailored to each patient's prosthetic design and accessibility and their manual dexterity.

### Multiple oral self-care interventions

Appropriate modes of at-home oral self-care are necessary to maintain peri-implant health. A multimodal approach combining a powered toothbrush, interproximal brushes or oral irrigators, and an evidence-supported dentifrice (e.g., triclosan-based) is most effective for long-term peri-implant maintenance (22). Oral health providers must understand the significance of patient home care and patient adherence to multiple interventions to deliver effective education and reinforce proper at-home techniques. Clinically, these findings emphasize the importance of a comprehensive, evidence-based home care routine for implant patients. The integration of multiple self-care interventions offers superior biofilm control compared to single-method approaches. Effective clinical implementation requires ongoing patient education, individualized product selection, and reinforcement of proper techniques during maintenance appointments to ensure patients can achieve optimal peri-implant tissue stability through consistent home care.

### Strengths and limitations

The strengths of this systematic review include reporting in accordance with the PRISMA 2020 statement (16) and the protocol was registered in the PROSPERO database (CRD420251028140). In addition, the search was designed by a health sciences librarian (NT-M). The limited number of articles (*n* = 12) in this systematic review is a limitation and reduces the generalizability of the outcomes. It is noteworthy that the first line of defense for peri-implant diseases and conditions is professional treatment interventions. The exclusion of studies that included professional treatment interventions (i.e., non-surgical scaling and root planing, periodontal maintenance, and prophylaxis) in conjunction with the intervention is a limitation. The exclusion criteria may have omitted studies that would have provided insights transferable to other populations and peri-implant diseases and conditions. The lack of studies on patient education for oral self-care, dietary interventions, and probiotics is also a limitation. These studies may have provided alternative interventions to reduce peri-implant diseases and conditions.

### Certainty of evidence

The overall certainty of the evidence in this systematic review was moderate to low.

The SR by Trombelli and Farina had a high risk of bias related to the number of research questions, limited key words, having only one screener, and having no information on minimizing error in data collection (21). The SR by Checchi et al. did not use a validated risk of bias tool and used the inclusion and exclusion criteria, which may have resulted in bias (22). The SR by Preda et al. only had one screener, which may have resulted in bias (23).

After applying the ROBIS tool, the SR by Gandhi et al. had the lowest risk of bias (24).

As for the certainty of evidence after applying RoB2, the study by Sreenivasan et al. had an overall low risk of bias (26). The remaining seven studies all demonstrated some concerns (27–33). These were largely related to domain 2, specifically, the participants being aware of their assigned intervention. However, it is important to note that in these specific studies (28–33), masking participants to their assigned intervention was not feasible. Two studies (29, 30) did not mention the extent of missing data or attrition; therefore, “no information” was documented for domain 3 and these studies were deemed to be of some concern. It was unclear in two studies (28, 30) whether the examiner was masked and this resulted in them being of some concern for domain 4. Two studies (29) provided no information about a pre-specified analysis plan and were deemed of some concern for domain 5. Despite the certainty of evidence based on the ROBIS tool and RoB2, this systematic review achieved the goal of itemizing the domains and categories for optimal home care devices, aids, and adjuncts for peri-implant diseases and conditions. Furthermore, this systematic review and its protocol were registered on PROSPERO, which did not indicate that the authors should be contacted for missing information to determine risk of bias.

### Future directions

Future clinical research should further investigate evidence-based oral self-care practices to support peri-implant health, while emphasizing patient education and awareness of peri-mucositis as a critical strategy for preventing peri-implantitis. Given the growing shortage of dental care providers in many regions, consistent access to professional implant maintenance may be limited. This makes effective home care even more essential, particularly for patients facing geographic, economic, or systemic barriers to care. Future systematic reviews should consider including studies with professional interventions and PICOT (PICO with the addition of timeframe) to determine whether the duration of the oral self-care intervention is a critical determinant of the intervention's effect or the outcome's relevance. In addition, future research should consider the patient’s history of periodontitis, the type of implant prosthesis, and the functional time of the implant. These criteria may provide beneficial evidence on best practices for oral self-care based on the stage of periodontitis, implant disease classification, and cleansing ability based on implant restoration type. Furthermore, the development and implementation of standardized clinical guidelines for both in-office implant maintenance and at-home oral hygiene instruction are necessary to promote consistency in care and optimize outcomes across diverse populations.

## Conclusion

Best oral self-care practices for effective prevention and management of peri-implant diseases should include a tailored regimen of triclosan-containing toothpaste, a powered/oscillating toothbrush, and an interdental aid (preferably interproximal brushes or oral irrigators). Chemical adjuncts, such as stannous fluoride/amine fluoride rinses, may offer additional anti-inflammatory benefits. Individualized oral self-care regimens, regular professional maintenance, and ongoing patient education remain fundamental components of long-term peri-implant health.

## Data Availability

The original contributions presented in the study are included in the article/Supplementary Material, further inquiries can be directed to the corresponding author.

## References

[1] Gaviria L Salcido JP Guda T Ong JL. Current trends in dental implants. J Korean Assoc Oral Maxillofac Surg. (2014) 40(2):50–60. 10.5125/jkaoms.2014.40.2.50 24868501 PMC4028797

[2] Elani HW Starr JR Da Silva JD Gallucci GO. Trends in dental implant use in the U.S., 1999–2016, and projections to 2026. J Dent Res. (2018) 97:1424–30. 10.1177/0022034518792567 [Epub ahead of print]. 30075090 PMC6854267

[3] Buser D Sennerby L De Bruyn H. Modern implant dentistry based on osseointegration: 50 years of progress, current trends and open questions. Periodontol 2000. (2017) 73(1):7–21. 10.1111/prd.12185 28000280

[4] Albrektsson T Buser D Sennerby L. Crestal bone loss and oral implants. Clin Implant Dent Relat Res. (2012) 14(6):783–91. 10.1111/cid.12013 23199435

[5] Lee CT Huang YW Zhu L Weltman R. Prevalences of peri-implantitis and peri-implant mucositis: systematic review and meta-analysis. J Dent. (2017) 62:1–12. 10.1016/j.jdent.2017.04.011 28478213

[6] Zitzmann NU Berglundh T. Definition and prevalence of peri-implant diseases. J Clin Periodontol. (2008) 35(8 Suppl):286–91. 10.1111/j.1600-051X.2008.01274.x 18724856

[7] Diaz P Gonzalo E Gil Villagra LJ Miegimolle B Suarez MJ. What is the prevalence of peri-implantitis? A systematic review and meta-analysis. BMC Oral Health. (2022) 22(1):449. 10.1186/s12903-022-02493-8 36261829 PMC9583568

[8] Howe MS Keys W Richards D. Long-term (10-year) dental implant survival: a systematic review and sensitivity meta-analysis. J Dent. (2019) 84:9–21. 10.1016/j.jdent.2019.03.008 30904559

[9] Caton JG Armitage G Berglundh T Chapple IL Jepsen S Kornman KS A new classification scheme for periodontal and peri-implant diseases and conditions—introduction and key changes from the 1999 classification. J Periodontol. (2018) 89(Suppl 1):S1–8. 10.1002/JPER.18-0157 29926946

[10] Berglundh T Armitage G Araujo MG Avila-Ortiz G Blanco J Camargo PM Peri-implant diseases and conditions: consensus report of workgroup 4 of the 2017 world workshop on the classification of periodontal and peri-implant diseases and conditions. J Periodontol. (2018) 89(Suppl 1):S313–18. 10.1002/JPER.17-0739 29926955

[11] Araujo MG Lindhe J. Peri-implant health. J Clin Periodontol. (2018) 89(Suppl):S249–56. 10.1002/JPER.16-0424 29926494

[12] Renvert S Persson GR Pirih FQ Camargo PM. Peri-implant health, peri-implant mucositis, and peri-implantitis: case definitions and diagnostic considerations. J Periodontol. (2018) 89(Suppl 1):S304–12. 10.1002/jper.17-0588 29926953

[13] Heitz-Mayfield LJA Salvi GE. Peri-implant mucositis. J Periodontol. (2018) 89(Suppl 1):S257–66. 10.1002/JPER.16-0488 29926954

[14] Schwarz F Derks J Monje A Wang H-L. Peri-implantitis. J Periodontol. (2018) 89(Suppl 1):S267–90. 10.1002/JPER.16-0350 29926957

[15] Schwarz F Becker J Civale S Hazar D Iglhaut T Iglhaut G Onset, progression and resolution of experimental peri-implant mucositis at different abutment surfaces: a randomized controlled two-centre study. J Clin Periodontol. (2018) 45(4):471–83. 10.1111/jcpe.12868 29331021

[16] Page M McKenzie J Bossuyt P Boutron I Hoffmann TC Mulrow CD The PRISMA 2020 statement: an updated guideline for reporting systematic reviews. Br Med J. (2021) 372:71. 10.1136/bmj.n71 PMC8005924 33782057

[17] Eagle I Theis-Mahon N Arnett MC. Oral self-care for the prevention and management of peri-implant conditions and diseases: A systematic review. PROSPERO 2025 CRD420251028140. Available online at: https://www.crd.york.ac.uk/PROSPERO/view/CRD420251028140 (Accessed October 31, 2025). 10.3389/froh.2025.1657025 PMC12756408 41487703

[18] Miller S Forrest J. Enhancing your practice through evidence-based decision making: PICO, learning how to ask good questions. J Evid Based Dent Pract. (2001) 1:136–41. 10.1016/S1532-3382(01)70024-3

[19] Covidence Systematic Review Software. Melbourne, VIC, Australia: Veritas Health Innovation. Available online at: http://www.covidence.org (Accessed October 31, 2025).

[20] Whiting P Savović J Higgins JP Caldwell DM Reeves BC Shea B ROBIS: a new tool to assess risk of bias in systematic reviews was developed. J Clin Epidemiol. (2016) 69:225–34. 10.1016/j.jclinepi.2015.06.005 26092286 PMC4687950

[21] Trombelli L Farina R. Efficacy of triclosan-based toothpastes in the prevention and treatment of plaque induced periodontal and peri-implant diseases. Minerva Stomatol. (2013) 62(3):71–88. 23518778

[22] Checchi V Racca F Bencivenni D Bianco LL. Role of dental implant homecare in mucositis and peri-implantitis prevention: a literature overview. Open Dent J. (2019) 13:470–7. 10.2174/1874210601913010470

[23] Preda C Butera A Pelle S Pautasso E Chiesa A Esposito F The efficacy of powered oscillating heads vs. powered sonic action heads toothbrushes to maintain periodontal and peri-implant health: a narrative review. Int J Environ Res Public Health. (2021) 18(4):1468. 10.3390/ijerph18041468 33557327 PMC7915098

[24] Gandhi G Masanam BSL Nair AS Semani N Chopra A Ramanarayanan V. Efficacy of oral irrigators compared to other interdental aids for managing peri-implant diseases: a systematic review. BDJ Open. (2025) 11(1):7. 10.1038/s41405-025-00301-3 39880827 PMC11779913

[25] Minozzi S Cinquini M Gianola S Gonzalez-Lorenzo M Banzi R. The revised Cochrane risk of bias tool for randomized trials (RoB 2) showed low interrater reliability and challenges in its application. J Clinical Epidemiol. (2020) 126:37–44. 10.1016/j.jclinepi.2020.06.015 32562833

[26] Sreenivasan PK Vered Y Zini A Mann J Kolog H Steinberg D A 6-month study of the effects of 0.3% triclosan/copolymer dentifrice on dental implants. J Clin Periodontol. (2011) 38(1):33–42. 10.1111/j.1600-051X.2010.01617.x 20831669

[27] Ramberg P Lindhe J Botticelli D Botticelli A. The effect of a triclosan dentifrice on mucositis in subjects with dental implants: a six-month clinical study. J Clin Dent. (2009) 20(3):103–7. 19711612

[28] Allocca G Pudylyk D Signorino F Grossi GB Maiorana C. Effectiveness and compliance of an oscillating-rotating toothbrush in patients with dental implants: a randomized clinical trial. Int J Implant Dent. (2018) 4(1):38. 10.1186/s40729-018-0150-6 30536124 PMC6286907

[29] Truhlar RS Morris HF Ochi S. The efficacy of a counter-rotational powered toothbrush in the maintenance of endosseous dental implants. J Am Dent Assoc. (2000) 131(1):101–7. 10.14219/jada.archive.2000.0028 10649881

[30] Di Carlo F Quaranta A Di Alberti L Ronconi LF Quaranta M Piattelli A. Influence of amine fluoride/stannous fluoride mouthwashes with and without chlorhexidine on secretion of proinflammatory molecules by peri-implant crevicular fluid cells. Minerva Stomatol. (2008) 57(5):215–21; 221–5. 18496484

[31] Mahajani MJ Kalla M Sonkesriya S Mehra P Laddha R Akade G. Comparison of the impact of two interdental cleaning devices on the reduction of bleeding around implants. J Pharm Bioall Sci. (2024) 16:S192–5. 10.4103/jpbs.jpbs_453_23 PMC11001108 38595601

[32] Magnuson B Harsono M Stark PC Lyle D Kugel G Perry R. Comparison of the effect of two interdental cleaning devices around implants on the reduction of bleeding: a 30-day randomized clinical trial. Compend Contin Educ Dent. (2013) 34(Spec No 8):2–7. 24568169

[33] AlMoharib HS AlAskar MH Abuthera EA Alshalhoub KA BinRokan FK AlQahtani NS. Efficacy of three interdental cleaning methods for peri-implant health maintenance of single implant supported crowns: a randomised clinical trial. Oral Health Prev Dent. (2024) 22:51–6. 38223961 10.3290/j.ohpd.b4854607 PMC11619878

[34] Ribeiro FV Casati MZ Casarin RC Corrêa MG Cirano FR Negri BM Impact of a triclosan-containing toothpaste during the progression of experimental peri-implant mucositis: clinical parameters and local pattern of osteo-immunoinflammatory mediators in peri-implant fluid. J Periodontol. (2018) 89(2):203–12. 10.1002/JPER.17-0302 29520826

[35] Kozlovsky A Goldberg S Natour I Rogatky-Gat A Rogatky-Gat A Gelernter I Efficacy of a 2-phase oil: water mouthrinse in controlling oral malodor, gingivitis, and plaque. J Periodontol. (1996) 67(6):577–82. 10.1902/jop.1996.67.6.577 8794967

